# Altered Expression of hsa_circ_0001445 and hsa_circ_0020397 in Breast Cancer Representing Associations with BMI and Reproductive Factors

**DOI:** 10.34172/aim.2022.127

**Published:** 2022-12-01

**Authors:** Zahra Firoozi, Elham Mohammadisoleimani, Hassan Dastsooz, Abdolreza Daraei, Seyed Alireza Dastgheib, Atefeh Raoofat, Hosein Mansoori, Yaser Mansoori, Mohammad Mehdi Naghizadeh

**Affiliations:** ^1^Department of Medical Genetics, Fasa University of Medical Sciences, Fasa, Iran; ^2^Department of Medical Biotechnology, Fasa University of Medical Sciences, Fasa, Iran; ^3^IIGM-Italian Institute for Genomic Medicine, c/o IRCCS, Candiolo, Torino, Italy; ^4^Candiolo Cancer Institute, FPO-IRCCS, Candiolo Cancer (IT), Torino, Italy; ^5^Department of Life Sciences and Systems Biology, University of Turin, Via Accademia Albertina, Turin, Italy; ^6^Department of Medical Genetics, School of Medicine, Babol University of Medical Sciences, Babol, Iran; ^7^Department of Medical Genetics, School of Medicine, Shiraz University of Medical Sciences, Shiraz, Iran; ^8^Noncommunicable Diseases Research Center, Fasa University of Medical Sciences, Fasa, Iran

**Keywords:** Breast Cancer, Circular RNAs, Hsa_circ_0001445, Hsa_circ_0020397

## Abstract

**Background:**

Circular RNAs (circRNAs), one of the recent subclasses of non-coding RNAs (ncRNAs), show pivotal functions in regulation of gene expression and have significant roles in malignancies including breast cancer (BC). This study was aimed to assess the hsa_circ_0001445 and hsa_circ_0020397 expression and role in BC, as well as the potential circRNA/miRNA/mRNA crosstalk in these contexts.

**Methods:**

The expression of hsa_circ_0001445 and hsa_circ_0020397 in 50 breast tumors and 50 normal tissues adjacent to the tumors was investigated using quantitative real-time polymerase chain reaction (qRT-PCR). Finally, bioinformatics analyses were used to uncover hsa_circ_0001445, hsa_circ_0020397-miRNA-mRNA potential regulatory networks.

**Results:**

The hsa_circ_0001445 expression was considerably downregulated in malignant tissues compared to their normal counterparts (*P*=0.020), while the hsa_circ_0020397 showed an upregulated pattern (*P*<0.001). Additionally, it was observed that the higher expression of hsa_circ_0001445 was associated with hair dye avoidance (*P*=0.034) and normal body mass index (BMI) (*P*=0.016) while hsa_circ_0020397 over-expression had an important association with a lack of vitamin D consumption (*P*=0.039). On the other hand, lower expression of hsa_circ_0001445 was significantly associated with age at menarche ˂14 years (*P*=0.027). Our study also revealed that the two circRNAs have potential ability to regulate key mRNAs and miRNAs in competing endogenous RNA (ceRNA) networks.

**Conclusion:**

It is suggested that hsa_circ_0001445 and hsa_circ_0020397 with two opposite roles may be involved in BC development through sponging some miRNAs regulating ceRNA networks. However, their molecular interactions should be validated by further functional studies.

## Introduction

 Circular RNAs (circRNAs), a new subclass of non-coding RNAs (ncRNAs) formed from pre-mRNA by backsplicing processes,^1^ are mainly categorized into three groups: exonic circRNAs (EcRNA),^2^ circular intronic RNAs (ciRNA)^3^ and exon-intron circRNAs (ElciRNAs).^4^ Numerous studies have revealed that circRNAs are involved in a variety of cancers through different mechanisms, such as regulating the function and synthesis of proteins, acting as transcriptional regulators,^4,5^ and repressing the miRNA activity and consequently, suppressing the effects of miRNAs on their target mRNAs.^6^ circRNAs are steadier for degeneration by exonucleases or RNase R than their linear forms and therefore, because of their high structural stability, they can be used as diagnostic and prognostic biomarkers in different cancers including breast cancer (BC).^7,8^

 BC is the most frequent cancer with the greatest rates of morbidity and mortality among women worldwide.^9^ So, it is essential to figure out how BC develops and progresses at the molecular level, to improve early detection and treatment of BC patients. circRNAs have demonstrated potential functions in various biological processes of BC, including carcinogenesis, metastasis and chemoresistance through acting as miRNA sponges and subsequently, regulate the gene expression as a transcriptional regulator.^10,11^ In addition, previous studies have underlined the oncogenic or anti-oncogenic roles of circRNAs in BC as well as providing a new aspect for the treatment and prognosis of patients.^12^ Huang et al discovered that hsa_circ_0008039 promotes proliferation and invasion of BC cells by sponging miR-515-5p and subsequently upregulating CBX4.^13^ Another study has reported that circVAPA sponges miR-130a-5p and regulates BC cell migration and invasion.^14^

 hsa_circ_0001445 (circ-SMARCA5) has been recently identified to be downregulated in several types of solid tumors, including cervical cancer, hepatocellular carcinoma, gastric cancer, glioblastoma multiform, non-small cell lung cancer and BC, while it was up-regulated in prostate cancer.^15-21^ This circRNA is an exonic transcript of SMARCA5 gene located on chromosome 4.^22^ Although the expression of hsa_circ_0001445 in BC was studied by Xu et al, the association between its expression and demographic and clinicopathological characteristics of patients with BC as well as the circRNA-miRNA-mRNA network for this circRNA is unknown yet.

 hsa_circ_0020397 is an EcRNA derived from the protein-coding gene dedicator of cytokinesis 1 (DOCK1) and is located on chromosome 10.^22^ This circRNA is overexpressed in colorectal cancer (CRC) cells and may have a role via influencing the function of miR-138. hsa_circ_0020397 acts as miR-138 sponge that results in suppressing this miRNA function and consequently, PD-L1 and TERT, two miR-138 target genes, are up-regulated. It is suggested that hsa_circ_0020397/miR‐138/PD-L1, TERT axis contributes to modulating cell viability, apoptosis, and invasion of CRC.^23^ As noted above, hsa_circ_0001445 and hsa_circ_0020397 could act as an oncogene or tumor suppressor gene in the various steps of carcinogenesis. However, the hsa_circ_0020397 expression and functions, and the hsa_circ_0001445 association with clinicopathological characteristics of patients remain unknown in BC patients.

 The purpose of this study is to examine the hsa_circ_0001445 and hsa_circ_0020397 expression in breast tumor tissues and their normal matched counterparts and to assess the relationship of their expression with the demographic and clinical aspects of BC patients. Some reports show that circRNAs could regulate the expression of mRNAs through miRNA sponging in competing endogenous RNA (ceRNA) networks.^24^ Therefore, in the current study, the putative circRNA-miRNA-mRNA axes for these two circRNAs in BC were investigated using bioinformatic techniques.

## Materials and Methods

###  Patients and Tissue Collection 

 We collected 50 tumors and 50 normal tissues adjacent to the tumors from the BC patients in Shahid Faghihi hospital, Shiraz, Iran. The patients had not received chemotherapy or radiotherapy prior to surgery. Written informed consent approved by the ethical committee at Fasa University of Medical Sciences (ethical code: IR.FUMS.REC.1398.187) was obtained from all patients. The demographic and reproductive characteristics of the BC patients are shown in Table 1 and Table 2.

**Table 1:** Association Between the Expression of Hsa_circ_0001445 and Hsa_circ_0020397 and Demographic and Pathological Characteristics of Studied Cases

**Characteristics**		**hsa_circ_0001445 level**					**hsa_circ_0020397 level**				
		**N**	**Mean**	**SD**	**Median**	***P***	**N**	**Mean**	**SD**	**Median**	***P***
Age	< 50	22	546.5	2552.7	0.748	0.969	22	378.6	1730.6	4.477	0.667
	≥ 50	28	1.3	1.5	0.635		28	13.9	24.1	3.670	
Tumor size	< 2.5	31	1.5	1.7	0.843	0.299	31	13.6	23.3	4.542	0.897
	≥ 2.5	19	632.4	2746.9	0.596		19	436.7	1862.2	3.018	
Estrogen receptor	Negative	1	6.3	0	6.267	0.119	1	18.1	0	18.107	0.315
	Positive	49	246.1	1710.6	0.661		49	177.5	1159.4	4.238	
Progesterone receptor	Negative	2	1.7	1.9	1.719	0.692	2	11.7	14.1	11.676	0.729
	Positive	48	251.2	1728.3	0.680		48	181.1	1171.4	4.253	
HER2	Negative	33	364.8	2084.3	0.661	0.886	33	257.8	1412.8	3.234	0.430
	Positive	17	1.3	1.7	0.843		17	12.3	14.8	5.651	
Nuclear grade	1	6	1.4	1.2	1.113	0.832	6	15.2	14.5	12.201	0.593
	2	36	334.6	1995.6	0.635		36	238.5	1352.4	3.822	
	3	8	0.8	0.5	0.652		8	4.9	4.5	3.736	
Metastasis to lymph nodes	Yes	15	2.7	7	0.306	0.144	15	5.7	6.3	1.776	0.130
	No	35	343.5	2024	0.713		35	246.6	1371.3	4.686	
Class of invasive carcinoma	ILC	1	0.7	0	0.713	0.917	1	2.8	0	2.845	0.775
	IDC	49	246.1	1710.6	0.661		49	177.8	1159.4	4.268	
Age at menopause	< 50	14	1.3	1.6	0.932	0.762	14	20.5	33.6	2.552	0.686
	≥ 50	21	1.5	1.5	0.661		21	10.9	12.8	4.542	
Menopausal status	Pre	15	800.7	3091.4	0.336	0.300	15	546.7	2096.9	4.268	0.560
	Post	35	1.4	1.6	0.713		35	14.8	23.5	4.238	
Age at menarche	< 14	31	1.7	4.9	0.562	**0.027**	31	9.1	14.1	3.103	0.171
	≥ 14	19	632.1	2746.9	1.551		19	443.9	1860.6	10.115	
Breastfeeding duration	0	6	5.2	10.9	0.447	0.909	6	19.6	23.7	9.590	0.818
	< 24	14	1.3	1.4	0.857		14	10.5	14.1	5.169	
	≥ 24	30	400.4	2186.2	0.602		30	281.7	1481.8	3.736	
Abortion history	No	36	1.8	4.6	0.579	0.235	36	13.2	22.8	4.253	0.713
	Yes	14	856.9	3200.2	1.316		14	588.7	2169.6	3.888	
Vitamin D consumption	No	25	2.2	5.4	0.797	0.308	25	18	26.5	5.651	**0.039**
	Yes	25	480.2	2394.9	0.506		25	330.7	16242	2.126	

IDC, invasive ductal carcinoma; ILC, invasive lobular carcinoma.

**Table 2:** Association Between the Expression of hsa_circ_0001445 and hsa_circ_0020397 and Demographic and Pathological Characteristics of Studied Cases, according to by Dividing Patients in to 2 Groups of High and Low Expressions

**Characteristics**		**hsa_circ_0001445 Level**					**hsa_circ_0020397 Level**				
		**Low**		**High**		***P***	**Low**		**High**		***P***
		**N**	**%**	**N**	**%**		**N**	**%**	**N**	**%**	
Age at menopause	< 50	5	36%	9	64%	0.332	8	57%	6	43%	0.581
	≥ 50	11	52%	10	48%		10	48%	11	52%	
Age at menarche	< 14	18	58%	13	42%	0.145	18	58%	13	42%	0.145
	≥ 14	7	37%	12	63%		7	37%	12	63%	
Number of pregnancies	≤ 3	18	50%	18	50%	1.000	17	47%	19	53%	0.529
	> 3	7	50%	7	50%		8	57%	6	43%	
OCP consumption	No	14	54%	12	46%	0.571	14	54%	12	46%	0.571
	Yes	11	46%	13	54%		11	46%	13	54%	
BMI	≤ 25	6	30 %	14	70%	**0.016** ^a^	8	40%	12	60%	0.230
	25-29	16	73%	6	27%		14	64%	8	36%	
	≥ 30	3	38%	5	62%		3	38%	5	62%	
Family history of cancer	No	13	52%	12	48%	0.777	13	52%	12	48%	0.777
	Yes	12	48%	13	52%		12	48%	13	52%	
Number of abortions	0	20	56%	16	44%	0.066	18	50%	18	50%	0.311
	1	3	25%	9	75%		5	42%	7	58%	
	> 1	2	100%	0	0%		2	100%	0	0%	
Breastfeeding duration	0	3	50%	3	50%	0.811	3	50%	3	50%	0.811
	< 24	6	43%	8	57%		6	43%	8	57%	
	≥ 24	16	53%	14	47%		16	53%	14	47%	
Hair dye use	No	2	20%	8	80%	**0.034**	4	40%	6	60%	0.480
	Yes	23	58%	17	42%		21	52%	19	48%	
Lymph node metastasis	Yes	9	60%	6	40%	0.355	10	67%	5	33%	0.123
	No	16	46%	19	54%		15	43%	20	57%	
Estrogen receptor	Negative	0	0 %	1	100%	0.312	0	0%	1	100%	0.312
	Positive	25	51%	24	49%		25	51%	24	49%	
Progesterone receptor	Negative	1	50%	1	50%	1.000	1	50%	1	50%	1.000
	Positive	24	50%	24	50%		24	50%	24	50%	
HER2	Negative	17	52%	16	48%	0.765	17	51%	16	49%	0.765
	Positive	8	47%	9	53%		8	47%	9	53%	

OCP, oral contraceptive pill; BMI, body mass index. ^a^ Generalized fisher exact test computes this *P* value as 0.019.

###  RNA Isolation and cDNA Synthesis assay

 Total RNA was isolated from the samples using the TriZol reagent (Invitrogen, Thermo Fisher). The concentration and quality of the extracted RNA was determined using spectrophotometer and agarose gel electrophoresis, respectively. Complementary DNA (cDNA) synthesis was performed by First strand cDNA synthesis kit (Thermo Scientific^TM^, Fermentas, Cat. No: K1622) according to the manufacturer’s recommendations.

###  Quantitative Real-Time Polymerase Chain Reaction Analysis 

 Real-time PCR was conducted in duplicate using circRNAs divergent primers and RealQ Plus 2x Master Mix Green with High ROX^TM^ (Ampliqon, Cat. No: A325402-25). β2M housekeeping gene was used for normalizing data. The primer sequences are listed in Table 3. Thermal cycling conditions were: 45 cycles of 95°C for 20 seconds, and then 60°C for 30 seconds. The 2 ^-∆∆CT^ (fold change) method was used to analyze the relative expression.

**Table 3:** Primer Sequences Used for qRT-PCR Assay

**Gene Symbol**	**Primer Sequences (5′ to 3′)**
hsa_circ_0001445 (F)	GAGAAAAACAAAAGGGAGGCTTG
hsa_circ_0001445 (R)	TCATCTCTGCAGTCTTCTTTGC
hsa_circ_0020397 (F)	GAGATTCTGAACTCATTCTTTTTATAACT
hsa_circ_0020397 (R)	GCAGGAAATATACCCTTCTTAGAC
β2M (F)	AGATGAGTATGCCTGCCGTG
β2M (R)	GCGGCATCTTCAAACCTCCA

###  Statistical Analysis

 ΔΔCt was calculated based on the mean and standard deviation, and fold change was defined using median. Wilcoxon test was performed to compare the fold changes between these samples. The relationship between circRNAs expression and clinicopathologic and demographic variables were analyzed by Mann–Whitney and Kruskal–Wallis tests. Based on the median, fold changes were grouped into high and low expression and comparison was made using the chi-square test. IBM SPSS 26 statistical software was used for data analyses. A statistically significant degree was defined as a *P* value less than 0.05.

###  In Silico Analyses

####  Construction of the ceRNA Network

 To construct the ceRNA network, we investigated miRNAs and mRNAs related to circRNAs. Circinteractome (https://circinteractome.nia.nih.gov) database was utilized for prediction of the interaction between circRNA and miRNA. miRNA_mRNA interaction was downloaded from the DIANA-miRPath (http://diana.imis.athena-innovation.gr/DianaTools/index.php?r=mirpath), TargetScan (http://www.targetscan.org/vert_72/) and mirTarBase (http://miRTarBase.mbc.nctu.edu.tw/) databases. The circRNA-miRNA-mRNA network was formed on the basis of circRNA-miRNA pairs, miRNA-mRNA pairs, and PPI (protein-protein interaction). The STRING database was applied to predict the protein-protein interaction (https://string-db.org). To construct and visualize the network, we then used the Cytoscape software (version 3.7.2).

###  Survival Analysis

 To assess gene survival analysis, we used the GEPIA (http://gepia.cancer-pku.cn/) database to evaluate gene survival analysis on the basis of gene expression and a log-rank test. For all genes involved in the two networks, survival analysis was performed.

###  Genes with Transcription Factors Role in These Networks

 The potential genes with transcription factor role were found through TF2DNA (http://fiserlab.org/tf2dna_db//search_genes), a database that contains detailed information of transcription factor binding motifs and the genes that they regulate.

## Results

###  hsa_circ_0001445 and hsa_circ_0020397 Expression in BC 

**Figure 1:**
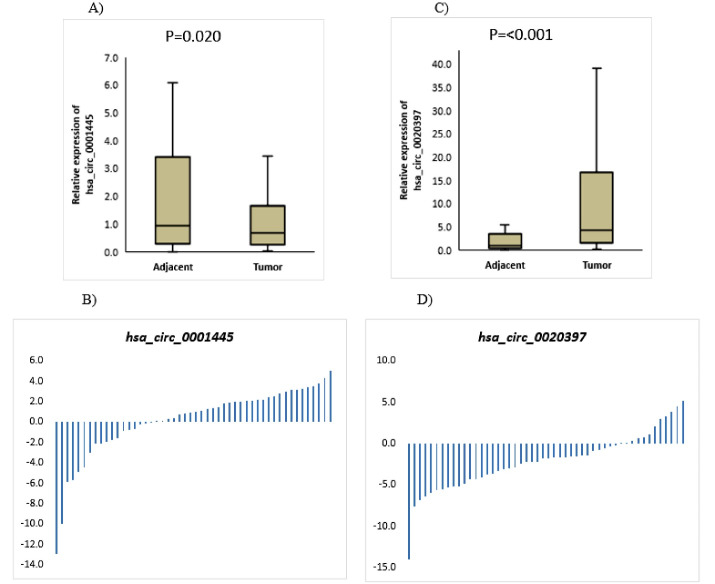
CircRNAs Expression Levels in Tumors Compared to Normal Adjacent Tissues. (A) hsa_circ_0001445 expression levels in tumors and their matched normal counterparts. (B) Scatter charts for comparing the hsa_circ_0001445 expression between tumor tissues and their matched normal ones, based on ΔΔCt.(C) hsa_circ_0020397 expression levels in tumor tissues and their matched normal ones. (D) Scatter charts for comparing the hsa_circ_0020397 expression between tumors and their normal tissues, based on ΔΔCt

 The circRNAs expression levels in tumor tissues and their matched tissues adjacent to the tumor were determined by qRT-PCR. The results of statistical analysis of qRT-PCR data revealed that the hsa_circ_0001445 expression was decreased in tumor tissue (Median = 0.680, Q1 = 0.251, Q3 = 1.648) compared to adjacent normal tissue (Median = 0.935, Q1 = 0.294, Q3 = 3.426), with a significant statistic *P*= 0.020 (Figure 1A and 1B); while hsa_circ_0020397 was significantly overexpressed in tumors (Median = 4.253, Q1 = 1.445, Q3 = 16.694) compared with paired adjacent normal tissues (Median = 0.956,Q1 = 0.357, Q3 = 3.511) (*P* < 0.001, Figure 1C and 1D).

###  Relationship Between the Expression of hsa_circ_0001445 and Demographic and Clinicopathological Features of Studied Patients 

**Figure 2:**
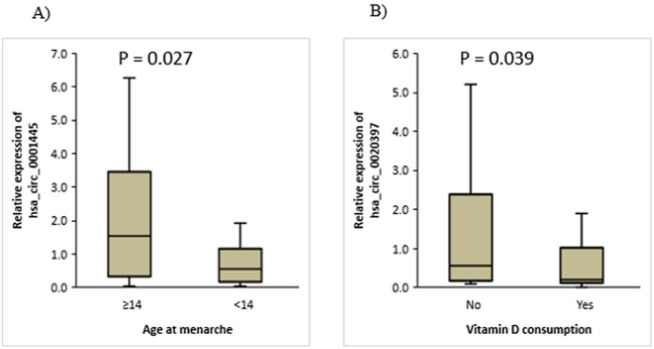
Relationship Between circRNAs Expression and Demographic Features Including Age at Menarche and Vitamin D Consumption.(A) Association between the expression of hsa_circ_0001445 and age at menarche. (B) Association between hsa_circ_0020397 expression and vitamin D consumption

 Our findings revealed a decrease in the expression level of hsa_circ_0001445 in tumor tissues of BC patients who had early menarche (age < 14 years, *P*= 0.027) (Figure 2A). These data are given in Table 1. The expression data of BC patients were also separated into high and low expression groups based on the median for hsa_circ_0001445. In patients with normal body mass index (BMI), gene expression was higher than in overweight and obese subgroups (*P*= 0.016), and the hsa_circ_0001445 expression was also high in people who did not use hair dye (*P*= 0.034) (Table 2).

###  Relationship Between hsa_circ_0020397 Expression and Demographic and Clinicopathological Features of Studied Patients 

 Our analyses showed that there was a significant association between vitamin D consumption and hsa_circ_0020397 expression levels, that the expression of this circRNA was higher in women who did not take vitamin D than those who took vitamin D (Figure 2B and Table 1), and this relation was statistically significant (*P*= 0.039).

###  Potential circRNAs -mediated Sponge Regulatory Network in Breast Cancer

**Figure 3:**
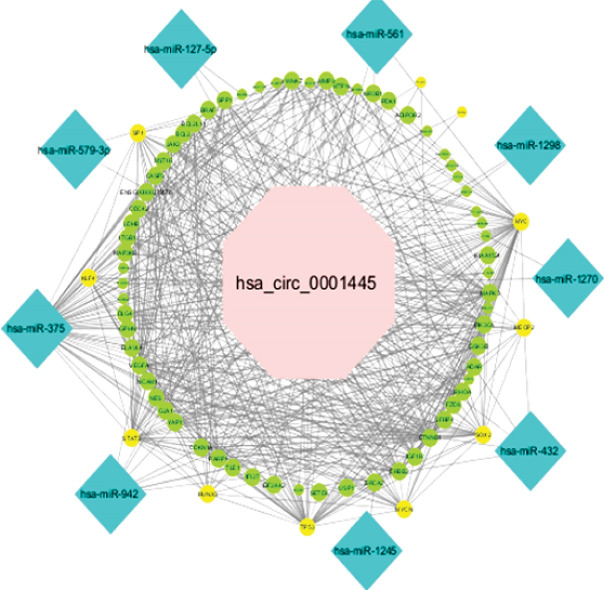
The Competing Endogenous RNA (ceRNA) Network of circRNA, miRNA, and mRNA. The pink octagon represents hsa_circ_0001445, the blue diamond nodes represent miRNA, and green ellipse nodes represent mRNA. The yellow ellipse nodes show mRNA members of the analyzed circRNA/miRNA/mRNA axis with the transcription factor role

**Figure 4:**
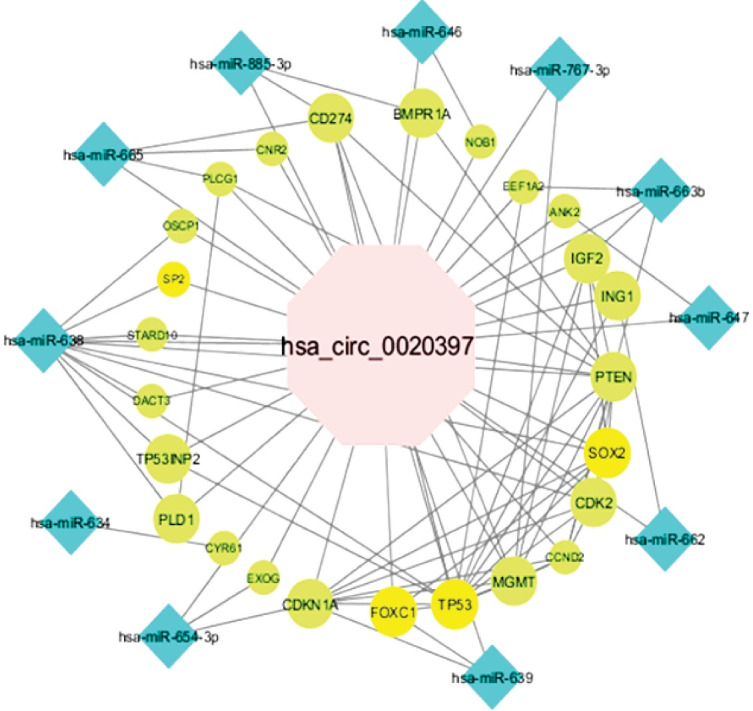
The hsa_circ_0020397 Mediated Sponge Regulatory Pathway in Breast Tumors. The pink octagon represents hsa_circ_0020397, whereas the blue diamond nodes indicate miRNAs and green ellipse represents mRNAs. The yellow ellipse nodes show transcription factor

 According to the *in silico* investigation results, the potential hsa_circ_0001445/miRNA/mRNA and hsa_circ_0020397/miRNA/mRNA network was generated. The hsa_circ_0001445/miRNA/mRNA axis was constructed on the basis of 1 circRNA, 9 miRNAs, and 73 mRNAs (Figure 3). The CASP3, SP1, CDC42, ITGB1, VEGFA, CDKN1A, YAP1, STAT3, TP53, ERBB2, IGF1R, MYCN, RHOA, SOX2, GSK3B, PIK3CA, MAPK3, MYC, MMP9, and YWHAZ had the most interactions among the mRNAs in this network as well as hsa-mir-375 and hsa-mir-942 with the most interactions among the miRNAs evaluated to regarding the network. This network contained 9, 73, and 418 pairs of circRNA-miRNA, miRNA-mRNA, and PPI, respectively. One circRNA, 11 miRNAs, and 23 mRNAs were found in the hsa_circ_0020397/miRNA/mRNA network (Figure 4). This network is composed of 11, 26, and 33 pairs of circRNA-miRNA, miRNA-mRNA, and PPI pairs, respectively. PTEN, TP53, SOX2, CDKN1A, MGMT, IGF2, CDK2, and CCND2 had the most interactions among mRNAs and hsa-mir-638 and hsa-mir-665 among miRNAs in this network.

###  The mRNA Member with Transcription Factor Function Identified in the Examined circRNA /miRNA/mRNA Networks 

 The mRNAs found in the hsa_circ_0001445/miRNA/mRNA network with the role of transcription factors included PLAG1, SP1, TP53, SOX2, STAT3, RUNX3, MYCN, KLF4, MECP2, and MYC. Furthermore, in the hsa_circ_0020397/miRNA/mRNA axis, FOXOC1, SOX2, SP2, and TP53 were the mRNAs with the role of transcription factor.

###  Survival Analysis

**Figure 5:**
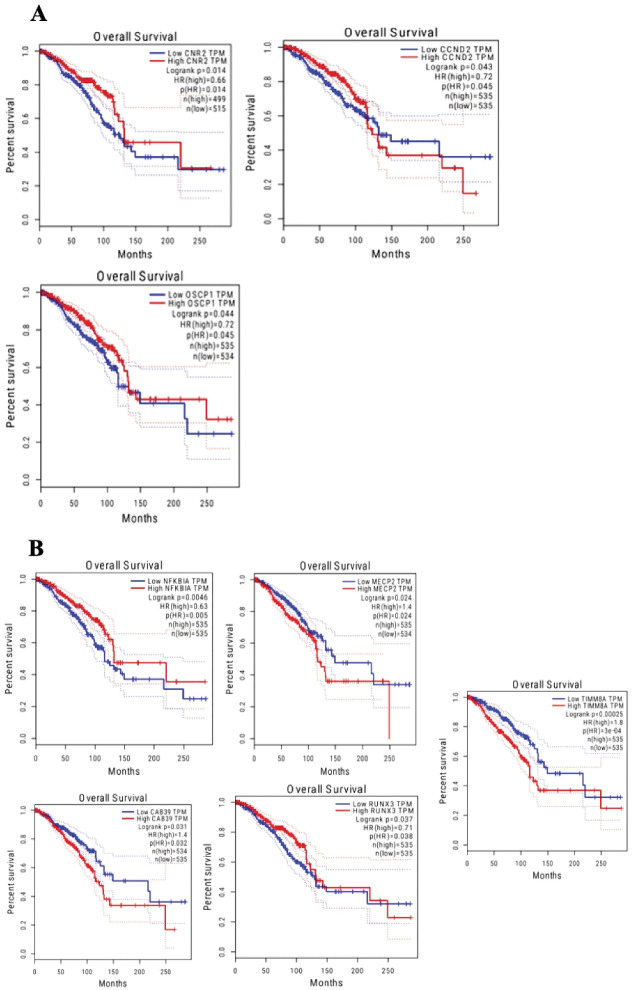
The mRNAs with Significant Differences in Survival Analysis and Transcription Factor Roles in The Examined circRNA/miRNA/mRNA Networks. (A) CNR2, CCND2 and OSCP1 mRNAs with the transcription factor function in the hsa_circ_0020397/miRNA/mRNA network. (B) NFKBIA, MECP2, CAB39, RUNX3 and TIMM8A mRNAs with the transcription factor function in hsa_circ_0001445/miRNA/mRNA network. The log-rank test was used and *P* value < 0.05 was selected as the threshold for survival prognosis significance

 Our bioinformatic analysis for the survival of all the genes using GEPIA web server revealed that in the hsa_circ_0020397/miRNA/mRNA network, CNR2, CCND2 and, OSCP1 showed notable increased expression in BRCA (log-rank P-values of 0.014, 0.043, and 0.044, respectively) compared to patients with lower expression levels (Figure 5A). Furthermore, the mRNAs with significant differences at survival analysis in the hsa_circ_0001445/miRNA/mRNA network included NFKBIA (log-rank *P* = 0.005), MECP2 (log-rank *P* = 0.024), CAB39 (log-rank *P*= 0.031), RUNX3 (log-rank *P* = 0.037), and TIMM8A (log-rank *P* < 0.001) (Figure 5B).

## Discussion

 Nowadays, microarray analysis and high throughput RNA sequencing data, have identified and characterized different classes of ncRNAs. circRNAs could act as an oncogene or tumor suppressor gene in various types of cancers, including BC, through regulating gene expression at transcriptional levels, acting as ceRNAs, and miRNA and protein sponge. There is growing evidence that dysregulation of these ncRNAs is associated with breast tumor initiation,^4-6^ development and progression, as well as their value to become potential novel biomarkers in BC.^8,13,14^ In our study, while a significant decrease in the hsa_circ_0001445 expression was identified in BC, hsa_circ_0020397 showed significantly increased expression in these tumor tissues. Some studies have addressed the roles of these two circRNAs in various malignancies.^15-21,23^ In cervical cancer, hepatocellular carcinoma, gastric cancer, glioblastoma multiforme, and non-small cell lung cancer, hsa_circ_0001445 (circSMARCA5) is down-expressed in malignant tissues as a tumor suppressor gene, while it is up-regulated in prostate cancer. It is demonstrated that hsa_circ_0001445 could regulate cell proliferation, invasion and migration in these cancers through the circRNA/miRNA/mRNA axis.^15-20^ Xu et al evaluated the hsa_circ_0001445 expression in 24 paired breast tumors and their matched non-cancerous samples which showed a down-expression in tumors; furthermore, they indicated that hsa_circ_0001445 could result in decreased expression of its parent gene, SMARCA5, by forming R-loops with it.^21^ Here, we assessed the expression of this circRNAs in 50 pairs of breast tumors and tumor’s adjacent normal tissues, and our results were consistent with the mentioned study. In addition, bioinformatically, the function of this circRNA in BC is shown by constructing a circRNA-miRNA-mRNA network on the basis of competitive endogenous RNA. hsa_circ_0001445 has been reported to sponge some miRNAs (hsa-mir-375, hsa-mir-942) through ceRNA networks to modulate its target mRNAs such as CASP3, SP1, CDC42, ITGB1, VEGFA, CDKN1A, YAP1, STAT3, TP53, ERBB2, IGF1R, MYCN, RHOA, SOX2, GSK3B, PIK3CA, MAPK3, MYC, MMP9, and YWHAZ. In addition, the functional role of all miRNAs and mRNAs in BC has been previously investigated.

 The reported data revealed that hsa_circ_0020397 could function as an oncogene in CRC, similar to our results in BC. Additionally, hsa_circ_0020397 could play its function by inhibiting the role of miR-138, resulting in greater expression of TERT and PD-L1, and subsequently regulating the apoptosis, invasion and viability of CRC.^23^

 Bioinformatic analyses of the ceRNA network related to hsa_circ_0020397 were also examined. Therefore, by determining the role of miRNAs (hsa-mir-638, hsa-mir-665) and mRNAs (PTEN, TP53, SOX2, CDKN1A, MGMT, IGF2, CDK2, CCND2) involved in this network, it is suggested that hsa_circ_0020397 may have a major function in BC. Following *in-silico* studies and realizing the importance of genes involved in the network, genes with the role of transcription factor (TF) are distinguished, as illustrated in Figure 4 and Figure 5A.

 On a different level, this study revealed that patients with early-onset menarche (˂14 years) have much decreased expression of the hsa_circ_0001445 gene in their tumors. Early age at menarche is considered as an increased risk of developing BC through enhancing the frequency of ovulatory circles, and hence this demographic factor results in greater exposure to ovarian hormones, estrogens and progesterone, in a lifetime.^25,26^ These hormones are strongly implicated in breast cells growth and development, which may lead to increased risk of BC.^27^ Therefore, this finding suggests that one of the mechanisms of increased risk of BC with earlier age at menarche may be downregulation of hsa_circ_0001445, whose tumor suppressive role in BC was demonstrated in the present study.

 In the present study, our findings also revealed that patients with normal BMI showed significantly increased levels of the hsa_circ_0001445 expression than overweight and obese subgroups. It is well-established that high BMI increases the BC risk in postmenopausal women, and the possible explanation in such situations is increased levels of circulating estrogens, and decreased circulation of sex hormone binding globulin.^28^ Furthermore, after menopause, conversion of adrenal androgens to estrogens in adipose tissues is the main source of estrogens. Therefore, overweight and obesity elevate the exposure of the breast to estrogens, that may lead to greater breast proliferation.^29,30^ It has been suggested that ncRNAs are involved in the physiological processes of obesity in BC patients,^26,31^ and circRNAs may also contribute to the pathogenesis and development of obesity.^32^ On the other hand, since miRNAs could be involved in adipogenesis and obesity processes and lipid metabolism, it seems that circRNAs, which bind miRNAs and subsequently, upregulate their target genes, may be involved in the regulation of adipogenic differentiation and lipid metabolism^32-34^; for example, circRNA_11897 could act as miR-27a sponge and consequently result in regulation of adipocyte lipid metabolism.^32,35^ This study presents the first evidence for a link between the hsa_circ_0001445 expression and obesity in BC, but more studies are needed for further investigation.

 In the current study, the hsa_circ_0020397 expression was considerably related to vitamin D consumption, and high expression was observed in patients who did not consume vitamin D. Vitamin D is a steroid hormone, and the precursor of 1,25-dihydroxyvitamin D1 (25OH2D), which is involved in anti-cancer processes including induction of apoptosis and cell differentiation, and inhibition of cell proliferation, angiogenesis, invasion and metastasis.^36^ The interaction between vitamin D and its receptor, vitamin D receptor (VDR), also increases the expression of apoptotic genes and induces apoptosis.^37^ As VDR is found in epithelial breast cells, the role of vitamin D in BC has been suggested.^38^ Vitamin D could control the growth of normal breast cells, and also stop the growth of cancer cells.^39^ In addition, tumor cells in BC lose their ability to form the active metabolite of vitamin D, while their ability of degrading this hormone was increased.^40^ Moreover, GO enrichment analysis have documented the roles of circRNAs in vitamin D and VDR processes.^41^ Therefore, our findings may indicate a key clue to the molecular link between hsa_circ_0020397 and vitamin D intake associated with BC initiation and progression in women due to regulating vitamin D related pathways, but further studies are needed to prove it.

 Finally, we observed that the hsa_circ_0001445 expression was more elevated in BC patients who did not use hair dye than in participants who did. Endocrine-disrupting chemicals (EDC), aromatic amines, 4-aminobiphenyl (4-ABP) and p-Phenylenediamine found in many hair dyes, have been shown to be genotoxic and carcinogenic.^42,43^ These chemicals could reach the breast tissue and induce tumors in the mammary glands by producing mutations in the genome. 4-ABP has been suggested to have the ability to impair the estrogen-related pathways, and increase the BC risk.^44^ EDC could also stimulate breast tumorigenesis by hormonal dysregulation and affecting the endocrine system.^45^ Hair dye chemicals may also affect the expression of some miRNAs and consequently their target genes; for example, EDCs caused an increase in the expression of oncomiR, miR-21, in MCF-7 BC cell line, and a decrease in the expression of miR-21 target genes, PDCD4 and PTEN.^46^ Taken together, we suggest that EDCs could affect the expression of hsa_circ_0001445.

 It should be also noted that the sample size in this study is slightly small due to ethical issues; therefore, it would be difficult to identify more significant relationships from the data, and the larger the sample, the more precise the results will be.

 In conclusion, our findings highlighted that hsa_circ_0001445 which is significantly downregulated in BC and hsa_circ_0020397 which is overexpressed, could sponge off miRNAs and consequently result in up-expression of target mRNAs through circRNA-related ceRNA regulatory mechanisms in BC. Furthermore, as a pioneer in this field, we identified the remarkable relation between the hsa_circ_0001445 and hsa_circ_0020397 expression and some demographic characteristics in BC patients. However, more functional research is required to affirm our findings and to uncover the regulatory roles of these circRNAs in BC.
